# The complex interplay between weather, social activity, and COVID-19 in the US

**DOI:** 10.1016/j.ssmph.2023.101431

**Published:** 2023-05-28

**Authors:** Simone Ferro, Chiara Serra

**Affiliations:** a University of Milan, Italy; b Bocconi University, Italy

**Keywords:** COVID-19, Seasonality, Weather, Social activity, Mediation

## Abstract

Empirical studies on the impact of weather and policy interventions on Covid-19 infections have dedicated little attention to the mediation role of social activity. In this study, we combine mobile locations, weather, and COVID-19 data in a two-way fixed effects mediation model to estimate the impact of weather and policy interventions on the COVID-19 infection rate in the US before the availability of vaccines, disentangling their direct impact from the part of the effect that is mediated by the endogenous response of social activity. We show that, while temperature reduces viral infectiousness, it also increases the amount of time individuals spend out of home, which instead favours the spread of the virus. This second channel substantially attenuates the beneficial effect of temperature in curbing the spread of the virus, offsetting one-third of the potential seasonal fluctuations in the reproduction rate. The mediation role of social activity is particularly pronounced when viral incidence is low, and completely offsets the beneficial effect of temperature. Despite being significant predictors of social activity, wind speed and precipitation do not induce sufficient variation to affect infections. Our estimates also suggest that school closures and lockdowns are effective in reducing infections. We employ our estimates to quantify the seasonal variation in the reproduction rate stemming from weather seasonality in the US.

## Highlights

• Most studies on the COVID seasonality overlook the role of social activity.
• Social activity offsets one third of the beneficial effect of temperature.
• Social activity entirely offsets the effect of temperature when infections are low.
• Lockdowns and school closures are similarly effective in curbing infections.

## 1 Background

The COVID-19 outbreak has seen an unprecedented cooperative effort from the scientific community. Among the many research questions being addressed by epidemiologists and social scientists, the role of seasonal changes in weather on the spread of SARS-CoV-2 has received substantial attention.

Initially based on the behaviour of other viruses (Fares, 2013; Moriyama et al., 2020, among others) and then on ad-hoc lab experiments showing that SARS-CoV-2 is highly sensitive to temperature and UV radiation (see for instance Chan et al., 2020; Chin et al., 2020; He, Lau, et al., 2020; Ratnesar-Shumate et al., 2020), some early consensus was reached over the possibility that the warmer season might have substantially slowed down the spread of the virus, going in some cases as far as to predict its complete disappearance with summer. Excessive confidence in the beneficial role of temperature^1^ might have in some cases deterred or delayed the implementation of adequate policy interventions in the early phases of the pandemic.^2^

Indeed, differently from what one could have expected looking at laboratory experiments, observational studies trying to infer the role of weather conditions from real-world data (Bashir et al., 2020; Carleton et al., 2021; Carson et al., 2020; Liu et al., 2021; Menebo, 2020; Merow & Urban, 2020; Rendana, 2020; Tosepu et al., 2020; Wang et al., 2021; Xie & Zhu, 2020; Zhu et al., 2020; Şahin, 2020) have so far delivered conflicting evidence (see Kerr et al., 2021). While a final consensus on the true seasonality of the virus has not been reached, it is now established that high temperatures are not sufficient to prevent a rapid circulation of the virus.

To reconcile this with the results of laboratory experiments, one should consider that weather does not just affect the virus leaving everything else unchanged, but it also influences social activity, which in turn is a major determinant of the infection rate. Therefore, even perfect knowledge of all the relevant biological mechanisms would not be sufficient to predict seasonal changes in infections. To fully understand the role of weather in the evolution of contagion, the mediating role of social activity can not be overlooked.

While both the sensitivity of human behaviour to weather (see Cools et al., 2010; Liu et al., 2014) and the role of human behaviour on the spread of infectious diseases (see Adda, 2016; Badr et al., 2020; Cartenì et al., 2020; Dave et al., 2021; Glaeser et al., 2020) have been established, few studies have considered human mobility when investigating the role of weather in the COVID-19 pandemic (Ganslmeier et al., 2021; Shao et al., 2020; Wilson, 2020; Zhang et al., 2022). In this paper, we employ mediation analysis tools and a two-way fixed effects regression model to show that the beneficial effect of increased temperatures in reducing the infection rate is significantly attenuated by the mediation of social activity, and that the beneficial effect of temperature is completely offset by increased social activity when fewer cases are being detected. We contribute to the existing literature by estimating the separate components of the effects of interest (and their interplay) within a model which builds upon the causal mediation analysis methodology (Baron & Kenny, 1986; Heckman & Pinto, 2015; Imai et al., 2010).

In line with the literature on seasonal and pandemic influenza and coronaviruses (Moriyama et al., 2020), we quantify the effects of interest as changes in the reproduction number (Dietz, 1993). As the share of immune individuals is generally low in our setting, we interpret these effects as estimated changes in the *basic* reproduction number, *R*_0_.

To estimate the effect of weather on *R*_0_ without directly observing the daily number of infections,^3^ we first model the theoretical causal relationships between *R*_0_, weather conditions, and the mediator, as if we had complete information on the daily number of infections, and then we transform the model to approximate the data generating process and estimate the parameters of interest while taking into account the detection lag and possible changes in testing capacity. We exploit the quasi-random nature of weather to estimate its total effect on *R*_0_ through a two-way fixed effects model with Commuting-Zone (CZ henceforth) and date fixed effects, and we add controls for several potential confounders to account for the possible residual endogeneity in social activity.

We focus on the 625 CZs in the contiguous U.S. as several features make it a good setting for our purpose. First, the geographical extension and the presence of different climate regions induce substantial local independent variation in weather conditions. Second, no major policy intervention to limit individuals’ freedom of movement was implemented at the federal level. Finally, the estimated prevalence of the virus and the testing capacity are among the highest in the world. We focus on the period between the first detected case (20^*th*^ of January 2020) and the 13^*th*^ of December 2020, the day before the first COVID-19 jab was administered in the U.S.. This means that our findings refer to a population that is largely susceptible to the virus, and caution is required when extrapolating our findings to an immunised population.

The results of our analysis may contribute to a better understanding of the seasonal patterns of COVID-19 and viral diseases in general, as well as their responsiveness to changes in social activity and policy interventions, and thus improve our ability to forecast their future evolution and better target policy interventions. Furthermore, our findings may rationalise the conflicting evidence of the empirical studies on the effect of temperature that do not account for social activity.

## 2 Data and methods

### 2.1 Data

**Confirmed Cases -** Data on the daily cumulative number of COVID-19 cases are collected from state and local governments and health departments and distributed by the New York Times,.^4^ We collected the number of daily confirmed cases for each county and aggregated them at the CZ level.^5^ We excluded the reported anomalies (for example methodological changes, technical issues with the data system, delayed updates on national holidays) and we removed clear outliers, imputing a predicted value based on a linear approximation to the resulting gaps.

**Weather Data -** We collected weather information from the Copernicus Atmosphere Monitoring Service (CAMS).^6^ The ERA-5 Land database includes meteorological indicators interpolated on a 9 × 9 *km*^2^ grid. We assigned daily weather measures to each CZ based on the point of the grid that is closest to the centre of the CZ,^7^ restricting to the interval 8 a.m. - 10 p.m..

We considered three weather variables: precipitations (the fraction of rainy hours), wind speed (in meters per second), and temperature (in degree Celsius). All variables are measured at the daily level restricting to the interval from 8 a.m. to 10 p.m. (local time) under the assumption that most individual activities take place within this time window.

**Mobile Location Data -** We defined *social activities* as all the activities which involve direct interactions with other individuals, and we proxied their intensity with the average amount of time individuals spend out of their homes based on mobile location data from SafeGraph. One limitation is that mobile location data are not necessarily representative of the general population. In particular, older and non-white individuals are less likely to be represented in SafeGraph data (Coston et al., 2021). Another limitation of the use of this measure as a proxy for social activity is that some out-of-home activities do not involve social interactions, and not all social interactions take place out of home. Yet, for two individuals from different households to meet, at least one of the two must be observed out of home.

We computed two measures of social activity based on the physical location of a sample of tens of millions of anonymised mobile devices, collected and distributed by SafeGraph.^8^ The dataset includes daily information at the Census Block Group (CBG) level on the average time individuals spend out of their imputed sleeping locations, as well as on the observed number of daily visits to a rich sample of Points Of Interest (POI), including for instance major retail chains, local businesses, hotels, gyms, and parks. For each CZ, we computed the daily average time spent out of the (imputed) sleeping location,^9^ and the daily number of individual visits to indoor POI located within the CZ, classifying indoor locations based on their NAICS code, first, and then on the name of the POI.^10^ CZ averages were computed weighing each CBG by the number of active devices.^11^

**Non-Pharmaceutical Policy Interventions -** Data on non-pharmaceutical policy interventions are collected and distributed by the CoronaNet project (Cheng et al., 2020) which provides daily information on government policy actions taken in response to COVID-19. The data include start and end dates of different types of COVID-19 related policies for several countries, including the U.S..^12^ We considered policies implemented at any administrative level, imputing county-level policies to the corresponding CZs. We then aggregated the policies according to the macro area of intervention into five categories (Closure of Businesses or Government Services, Distancing, Masks, Lockdown, and School Closures).^13^

**Fig. 1:**
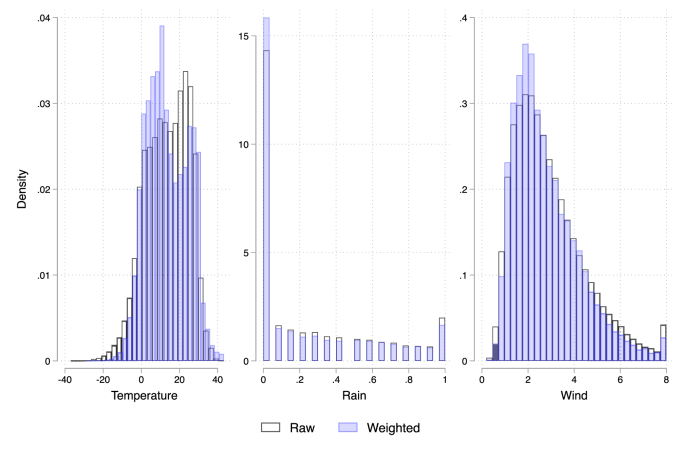
Distribution of Weather Variables Fig. 1: *Notes* - Sample densities, weights are proportional to the daily number of confirmed cases in the CZ-day cell. Temperature (degrees Celsius) and wind speed (meters per second) are computed as averages over the interval 8 a.m. - 10 p.m. (local time). Rain is the fraction of rainy hours (precipitation >0) over the same interval.

In Table 1, we report summary statistics for the main variables of interest, while in Fig. 1, we plot the sample distribution of the three main weather variables. As the identification of the effects of interest relies on the number of confirmed cases in the cell, we also report the distributions weighted by this quantity.

**Table 1:** Descriptive statistics.

	Mean	Std. Dev.	Min.	Max.	N
Temperature	14.094	10.948	−37.037	43.209	216,456
Rain	0.262	0.335	0	1	216,456
Wind	2.919	1.578	0.195	15.515	216,456
Av. Time Out of Home - Mediator A	3.286	0.647	1.141	6.41	203,580
Visits to Indoor Venues - Mediator B	1.142	0.3	0.114	5.481	196,560
Confirmed Cases - *N*_*c*,*t*_	76.3	319.3	0	23,213	214,695
CZ Population	518,090	1,285,296	890	18,629,045	229,198
Closure of Businesses or Government Services	0.49	0.5	0	1	215,280
Distancing	0.456	0.498	0	1	215,280
Masks	0.252	0.434	0	1	215,280
Lockdown	0.211	0.408	0	1	215,280
School Closure	0.314	0.464	0	1	215,280

Table 1: *Notes* - Temperature (degrees Celsius) and wind speed (meters per second) are computed as averages over the interval 8 a.m. - 10 p.m. (local time). Rain indicates the fraction of rainy hours (precipitation >0) over the same interval. Average Time Out of Home is measured in hours.

### 2.2 Mediation model

A mediation model describes how a third variable (the mediator) affects the relationship between two other variables (the independent variable and the dependent variable). Mediation models can be used to determine whether the effect of an independent variable on a dependent variable is fully or partially explained by the mediating variable.

**Fig. 2:**
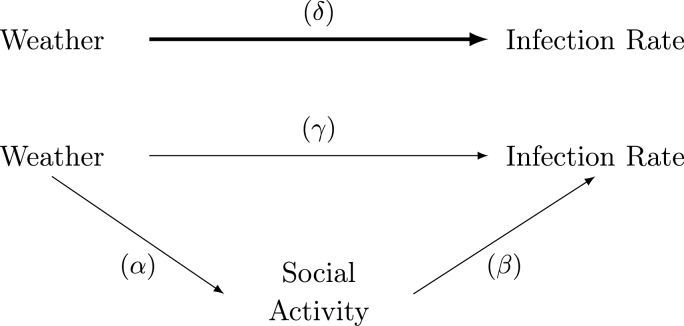
Dag representation.

In our setting, the total impact of weather conditions on the rate of infection (*δ* in Fig. 2) can be described as the result of two distinct mechanisms: a direct-biological effect of weather (*γ*), for instance on viral stability or hosts’ susceptibility, and an indirect impact (*α* × *β*), which can be further disentangled into the effect of weather on social activity (*α*), and the effect of social activity on the infection rate (*β*). In general, while the direct-biological component of the effect should predominantly depend on the chemical structure of the virus, its indirect part, and the total effect, are heavily context-dependent.

The objective of our empirical analysis is to quantify if, and to what extent, the local rate of infection responds to changes in weather conditions, and then to disaggregate the average treatment effect (*δ*) of each weather variable into two components: its direct effect, defined as the causal effect of weather holding the level of social activity fixed (*γ*), and its indirect component (*α* × *β*), that is transmitted by the endogenous response of social activity.

The main (unobserved) outcome of interest of our analysis is the daily reproduction number, the number of secondary infections associated with one single infectious individual in the CZ *c* in day *t* ($\frac{i_{c,t}}{C_{c,t}}$, where *i*_*c*,*t*_ is the number of individuals infected in the CZ *c* at time *t*, and *C*_*c*,*t*_ is the stock of contagious individuals in the cell). Notice that this quantity is very closely related to the reproduction rate *R*_0_, and that the effects on the former can be easily converted into effects on the latter by multiplying them by the assumed length of the infection period.

We assume that infections originate within the CZs in which they are detected. This assumption is necessary to model infections in a CZ as a function of the stock of infectious individuals, weather, and social activity in that CZ only.

According to the causal relations described by Fig. 2, infections are affected by weather conditions both directly (through *γ* in Equation (3)) and indirectly through their effect on social activity (through *α* in Equation (2)), which in turn affects infectiousness (through *β* in Equation (3)). Finally, *δ* is the total effect of weather (that includes the part that is transmitted by *S*_*c*,*t*_). More formally, the regression model we want to estimate can be summarised by the following three equations:

(1) $$\text{Total Effect: }\frac{i_{c,t}}{C_{c,t}}=\delta W_{c,t}+\psi X_{c,t}+u_{c,t}$$

(2) $$\text{Effect on the Mediator: }S_{c,t}=\alpha W_{c,t}+\psi^{\prime }X_{c,t}+u_{c,t}^{\prime }$$

(3) $$\text{Direct }+\text{ Mediated Effect: }\frac{i_{c,t}}{C_{c,t}}=\gamma W_{c,t}+\beta S_{c,t}+\rho S_{c,t}W_{c,t}+\psi^{\prime \prime }X_{c,t}+u_{c,t}^{\prime \prime }$$

Where *S*_*c*,*t*_ is a measure of social activity, *W*_*c*,*t*_ is a vector of weather conditions, and *X*_*c*,*t*_ is a vector of additional covariates.

Importantly, *X*_*c*,*t*_ includes both CZ and date fixed effects, which capture all the permanent geographical differences in terms of baseline health, socioeconomic factors and demographic characteristics of the different CZs, as well as all the events that may affect the evolution of the reproduction rate at the national level, thus characterising our regression model as a two-way fixed effects model. Hence, the residual variation in weather and social activity which contributes to the identification of the effects originates only from CZ-specific deviations from a common time trend.

Weather variables and social activity enter as second-order polynomials to capture possible concavity or convexity stemming from a nonlinear relationship between comfort and temperature, between temperature and viral infectiousness, and between social activity and viral infectiousness.^14^

To allow for possible interactions between weather and social activity,^15^ we followed the mediation analysis literature (see Imai et al., 2010) including interactions between each weather parameter and the mediator to Equation (3) in our preferred specification.

Consistent with the general structure of mediation analysis (Imai et al., 2010), the only difference between Equations (1), (3)) is the presence of *S*_*c*,*t*_ on the right-hand side (and the interaction between social activity and weather variables). Indeed, consistent with the interpretation of *δ* as the total effect and of *γ* as the direct effect of weather on infections, controlling for (or omitting) *S*_*c*,*t*_ serves the purpose of muting (or allowing) the causal path that runs through this variable.

To give a causal interpretation to the parameters of interest (*δ*, *α*, *β*, *γ*), we need to assume conditional exogeneity of the error terms in all the equations above, or *sequential ignorability* (Imai et al., 2010).

Given the quasi-random nature of weather conditions, the inclusion of CZ and date fixed effects in *X*_*c*,*t*_ is sufficient to ensure conditional exogeneity in Equations (1), (2)). For Equation (3), we need to control for all the factors that might simultaneously affect infections and social activity.

Still, there might be other confounding factors in Equation (3) that generate from local events and are thus not absorbed by fixed effects. We thus included a series of control variables to capture the main factors which might simultaneously affect social activity and the probability of infection at the sub-national level, and thus could confound our estimates if not appropriately accounted for. First, we included controls for the introduction of non-pharmaceutical interventions implemented at the state and local levels. Second, we included controls for the cumulative number of confirmed cases in the CZ as a share of the population and for the cumulative number of COVID-19 related deaths in the previous 14 days, which are expected to be the main drivers of the local level of awareness, fear of infection, and social norms on precautionary behaviours. Importantly, the share of positive cases in the CZ also proxies for the share of immunised individuals within the local population, which also may affect the local rate of infection.^16^

As the main dependent variable in the model is not directly observable, in order to estimate the parameters of interest it is necessary to express the model in terms of confirmed cases instead of infections. In Appendix A1, we show that, under mild assumptions on the data generating process, Equation (1) can be equivalently expressed as a function of observed quantities:

(4) $$\frac{N_{c,t}}{\sum_{j=1}^{L_{\max}}\left({\hat{p}}_{j}\hat{\varphi_{c,t-j}C_{c,t-j}}\right)}\approx \delta \frac{\sum_{j=1}^{L_{\max}}\left({\hat{p}}_{j}W_{c,t-j}\hat{\varphi_{c,t-j}C_{c,t-j}}\right)}{\sum_{j=1}^{L_{\max}}\left({\hat{p}}_{j}\hat{\varphi_{c,t-j}C_{c,t-j}}\right)}+\psi \frac{\sum_{j=1}^{L_{\max}}\left({\hat{p}}_{j}X_{c,t-j}^{*}\hat{\varphi_{c,t-j}C_{c,t-j}}\right)}{\sum_{j=1}^{L_{\max}}\left({\hat{p}}_{j}\hat{\varphi_{c,t-j}C_{c,t-j}}\right)}+\beta_{c}^{*}+\beta_{t}^{*}+\epsilon_{c,t}$$

where *N*_*c*,*t*_ is the number of COVID-19 cases officially confirmed in *t*; ${\hat{p}}_{j}$ is the probability that a positive individual is confirmed *j* days after the infection, conditional on the case ever being detected; $\hat{\varphi_{c,t-j}C_{c,t-j}}$ is the estimated stock of contagious individuals in the cell (*C*_*c*,*t*−*j*_) times the probability that a case is detected (*φ*_*c*,*t*−*j*_), which is allowed to vary both across CZs and over time; $\beta_{c}^{*}$ and $\beta_{t}^{*}$ are CZ-level and date-level fixed effects; and $X_{c,t-j}^{*}$ is a vector of covariates. The distribution of the detection lag ${\hat{p}}_{j}$, and the maximum assumed lag between infection and case confirmation *L*_max_, were modeled combining individual data on incubation time in China with symptoms-to-confirmation time in the U.S. as described in Appendix A1.

We estimated the parameters of interest through OLS regression, weighting observations according to the denominator of the dependent variable. To account for flexible serial and spatial correlation of the errors, we clustered errors both at the CZ level and at the date level. The same steps can be generalized to estimate the parameters in Equations (2), (3)).^17^

## 3 Results and discussion

Estimates of these parameters are reported in Tables A1, A2, and A3, where all coefficients have been rescaled to be converted from effects on the daily reproduction number to effects on *R*_0_ multiplying the estimates from the regression by the length of the assumed period of infectiousness. The mediating variable has been standardised so that, in the presence of interactions, the coefficients of each weather variable can be interpreted as the estimated effect when the level of the mediator is set to its mean level (0 in the standardised variable). As weather variables are not standardised, instead, the interactions between the mediator and weather complicate the interpretation of the coefficients on the former. Therefore, at the bottom of Table A3 we report the estimated coefficients on the mediator at median weather conditions.

**Fig. 3:**
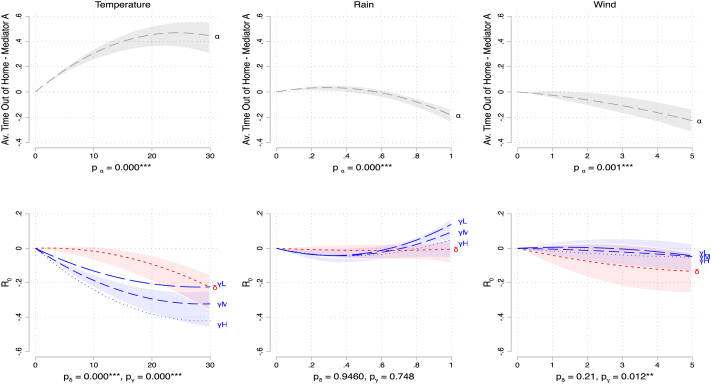
Estimated Total and Direct Effects of Weather on *R*_0_ Fig. 3: *Notes* - In the top panels, we plot the estimated effect of each weather variable on the mediator (*α*). Below, we plot the estimated total effect (*δ*) in red, together with the direct effect (*γ*) in blue, holding the level of the mediating variable fixed at three different levels: its mean level (*γM*), 0.5 SD below (*γL*), and 0.5 SD above (*γH*). The mediating variable is standardised. Each plot reports the corresponding 90% confidence bands, based on 1000 CZ-level block-bootstrap samples. Below each panel, we report the p-value of a joint significance F-test for the corresponding weather variable and its interactions (***p<0.01, **p<0.05, *p<0.1). See Column 4 of Tables A1, A2, and A3 for the complete results. (For interpretation of the references to colour in this figure legend, the reader is referred to the Web version of this article.)

To ease the interpretation of the estimates, we provide a visualisation of the results from our preferred specification (Column 4 of Tables A1, A2, and A3) plotting the estimated effects in Fig. 3. In the top panels, we plot *α*, the estimated effect of each weather variable on the (standardised) mediator, together with the corresponding 90% confidence bands (based on 1000 CZ-level block-bootstrap samples). In the panels below, we plot in red the estimated total effect *δ*, and in blue the estimated direct effect *γ*, the one that would be observed if the mediator was kept constant to a given level. As we allowed the direct effect of weather conditions to vary with the level of the mediator, the estimated direct effect is plotted holding the level of the mediating variable fixed at its mean level (*γ*_*M*_, 3 h and 17 min), and half a SD (or 19 min) below (*γ*_*L*_) and above (*γ*_*H*_) its mean. The values in the plot should be interpreted as the expected relative change in *R*_0_ when the x-variable increases from zero to the corresponding value on the x-axis.

Looking at the top-left panel of Fig. 3, the value of 0.44 at 30 °C implies that a change in temperature from 0 to 30° is estimated to increase the average time spent out of home by 44% of a standard deviation, or 17 min. Consistently with what can be considered a comfortable temperature range, the estimated profile is concave. The magnitude of this effect is sizeable, especially when compared to the effect of wind and rain.

The central blue line in the corresponding panel below, instead, shows that, if the mediating variable was held constant at its mean value, an increase in temperature from 0 to 30 °C would reduce *R*_0_ by 0.33. When the level of the mediator is allowed instead to vary endogenously (red line), the reproduction rate is estimated to drop by 0.22 following the same temperature increase. Hence, the endogenous response of social interaction is responsible for a 0.11 increase in *R*_0_.^18^ Overall, these figures suggest that the endogenous response of social activity substantially attenuates the beneficial effect of higher temperatures.

Consistently with the idea that the risk of infection is higher when social activity is more intense, the other two blue lines show that the negative direct effect of temperature on infections is larger (smaller) in absolute terms when the mediating variable is set to half of a standard deviation above (below) its mean.

Looking at the effect of rain and wind speed in the top panels of Fig. 3, we conclude that individuals are less active when precipitations and wind are more intense, but the estimated effects, although statistically significant, are substantially smaller than the effect of temperature. Consequently, in the panels below, the difference between the direct and the total effects is minor.^19^

A few other relevant conclusions can be drawn looking at the complete results reported in Tables A1, A2, and A3. First, after controlling for the cumulative share of confirmed cases and for recent deaths, the parameters of interest are very stable across specifications. Second, from Table A1 we conclude that the share of infected individuals is the most powerful predictor of the reproduction rate at the local. Results in Table A1 also suggest that lockdown policies and school closures are effective in curbing the spread of the virus. In particular, school closures are associated with a drop in the reproduction rate (−0.079) which is very similar to the effect of lockdown policies (−0.073). Although more research is needed to pinpoint the drivers of these effects, the fact that in our setting school closures are associated with a significant and sizeable drop in *R*_0_ might contribute to the current political debate on the costs and benefits of such regulations.

In Table A3, the same effects are estimated holding the level of the mediator variable fixed. Interestingly, the coefficient on lockdown is now substantially smaller and not significant. This, together with the 0.20 SD reduction in social activity associated with lockdown policies^20^ (see Table A2), is consistent with the fact that the effect of lockdowns can be almost entirely explained by their impact on the mediator, which also suggests that our measure of social activity is serving its purpose. The effect of school closures, instead, remains highly significant even controlling for the mediator, which suggests that only part of the effect of these policies can be explained by their impact on social activity and that other mechanisms contribute to the reduction of infections following school closures.

In order to understand to what extent the estimated negative direct effect of temperature on *R*_0_ is due to a biological impact, and to what extent this can instead be explained by lower indoor activity, we replicate the analysis employing the number of individual visits to *indoor* locations as an alternative mediator. Results (Figure A2 and Tables A4 and A5 in Appendix) suggest that temperature has an equally substantial positive impact on the number of visits to indoor locations, and that increasing temperature while keeping this alternative mediator fixed would reduce the reproduction rate by even more (0.42). This suggests that the distinction between indoor and outdoor activities does not play a crucial role in explaining our findings.

To investigate whether the relative importance of the different components of the effect depends on the epidemiological situation, we replicate our main specification splitting the sample according to the number of confirmed cases in the previous two weeks being above or below its median level. Results in Figure A3 and Tables A6, A7, and A8 in Appendix show that the mediation role of social activity is much more pronounced when the share of recently infected individuals is lower. In particular, while the endogenous response of social activity almost perfectly offsets the direct effect of temperature on *R*_0_ in the low-incidence sample, in the high-incidence sample the attenuation is barely detectable and the total impact of temperature is much stronger.

Our main conclusions are robust to other changes in the specification. In particular, we have replicated our estimates with an alternative infectiousness profile based on the estimated serial interval according to He, Tao, et al. (2020), and employing the 7-day moving average of new positive cases instead of the raw number. Results of these tests are reported in Figures A4 and A5 respectively and show the robustness of our conclusion to these alternative modelling choices. Furthermore, we have replicated our analysis computing weather indicators as 24-h averages instead of daytime averages, including and excluding quadratic terms and interactions between weather variables, and including alternative proxies of the observed severity of the epidemiological situation (cumulative number of confirmed cases instead than number of deaths).

## 4 Concluding remarks

Our estimates suggest that, if individuals’ behaviour did not respond to weather, a temperature increase from 0 to 30 °C would reduce the basic reproduction number by 0.33, but the potential beneficial effect of higher temperatures is substantially attenuated by an increase in social activity in warmer days, leading to a total effect of −0.22 when social activity is allowed to adjust to weather conditions. Similar conclusions can be reached when using the average number of visits to indoor venues as an alternative mediator. This suggests that reduced indoor activity can not explain our findings, and a biological effect of temperature on the virus is thus the most probable mechanism. A heterogeneous effect analysis suggests that the endogenous response of social activity is particularly pronounced when the COVID-19 incidence in the population is lower, leading to an almost negligible total effect of temperature.

**Fig. 4:**
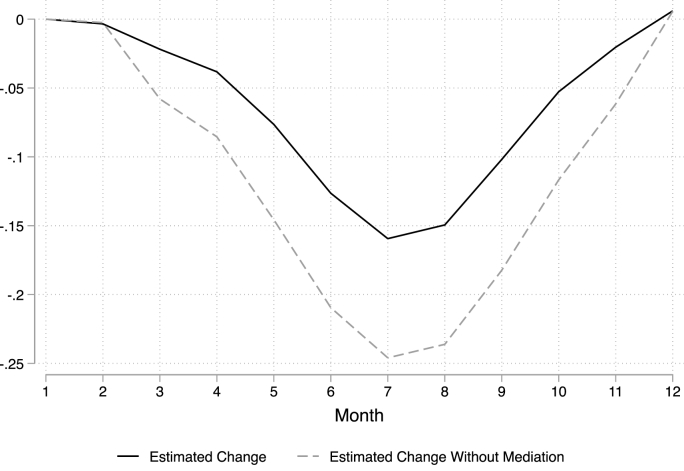
Effect of Seasonal Changes of Weather on *R*_0_, With and Without Mediation Fig. 4: *Notes* - Predicted seasonal changes in *R*_0_ are computed applying the point estimates of the coefficients in Column 5 of Tables A3 (without mediation) and in Column 4 of Table A1 (with mediation) to the observed weather conditions in each CZ-date and aggregating this quantity at the national level weighing each CZ by its population.

To illustrate the implications of our findings for the seasonal evolution of the reproduction rate, in Fig. 4 we plot the predicted relative change in *R*_0_ based only on the observed weather in the year 2020 with and without the part of the effect that is transmitted by the endogenous response of social activity. The figure suggests that if individuals did not respond to weather conditions by spending more time out of their home in the warmer season, the reproduction rate in the U.S. would drop by 0.25 in July with respect to January, whilst once the endogenous response of social activity is taken into account, this reduction is substantially attenuated (0.16).

Overall, this paper puts the role of temperature on the *number* of cases in perspective. Yet, if the effect of temperature on social activity varies with individual characteristics, the combined effect of the two competing mechanisms may result in substantial seasonal *compositional changes* of infections in terms of these characteristics, inducing seasonal changes in case fatality and hospitalisation rates. If, for instance, younger individuals are responsible for the observed increase in social activity on warmer days, then the mechanism may rationalise possible seasonal changes in the average age of infected individuals.

Our estimates also suggest that school closures and lockdown policies are similarly effective in reducing the reproduction rate. We therefore indirectly contribute to the strand of literature looking at the effectiveness of different policy interventions in reducing the spread of the virus (see for instance Friedson et al., 2021). In particular, in detecting a significant reduction in the reproduction rate after school closures in the U.S., our findings are in line with studies conducted in the same setting (Chernozhukov et al., 2021; Goldhaber et al., 2022) and suggest that caution should be taken before generalising the opposite conclusion reached by empirical studies conducted in different settings characterised by stricter containment measures (Gandini et al., 2021; Isphording et al., 2021, for instance).

When generalising our findings to other settings, one should keep in mind that they may vary depending on the specific geographical, socio-economic, and regulatory context. In particular, while we have no particular reason to expect the direct (biological) effect to vary depending on the setting, the specific mediation role of individuals’ behaviour may be limited in countries with more stringent distancing measures. Yet, regardless of the context, our findings suggest that any empirical analysis on the seasonality of viral diseases should not overlook the fundamental mediation role of social activity.

## Author statement

Simone Ferro and Chiara Serra equally contributed to the design and implementation of the research, to the analysis of the results and to the writing of the manuscript.

## Ethical statement

We confirm that the manuscript “The Complex Interplay between Weather, Social Activity, and COVID-19 in the US” is the authors' own original work, has not been previously published elsewhere and is not currently being considered for publication elsewhere.

## Declaration of competing interest

None.

## Data Availability

The authors do not have permission to share data.

## Footnotes

1 See Carlson et al. (2020) and Zaitchik et al. (2020) for a complete account of the misconceptions about how weather affects the transmission of COVID-19.

2 “Looks like by April, you know, in theory, when it gets a little warmer, it miraculously goes away.” D. Trump, 10th February 2020. “The virus doesn’t spread as fast in warm climates like ours”. J. Bolsonaro, 18th March 2020.

3 The most common solutions adopted by epidemiologists to estimate the reproduction rate are based on Bayesian approaches and implicitly assume some time-persistence in *R*_0_ (Bettencourt & Ribeiro, 2008). These assumptions are convenient when the objective is to monitor the evolution of an epidemic. Yet, they might be unsuitable when the focus of the analysis is on high-frequency variations in the infection rate.

4 The New York Times. (2021). Coronavirus (COVID-19) Data in the United States. Retrieved 15/01/2021, from https://github.com/nytimes/covid-19-data.

5 We used the geographical delineations revised by Fowler et al. (2016). More information and the crosswalk between counties and CZs available at https://sites.psu.edu/psucz/data/.

6 See Muñoz Sabater (2019).

7 We computed this as a population-weighted average of the longitudes and latitudes of the centroids of the census block groups within each CZ.

8 These data are based on the physical location of mobile devices over time, regardless of the mean of transportation.

9 More precisely, SafeGraph reports different measures relating devices to their “home”, where “home” is defined as the common nighttime location for the device over the past 6 weeks period. For our purpose, this may be problematic whenever an individual does not spend the night at the imputed location. To solve this issue, we employed the “bucketed time spent at the imputed home” to compute the average time spent away restricting to the individuals spending at least 8 h at their imputed home. As a substantial share of individuals are never observed at their imputed location, this restriction drastically reduces the estimated average amount of time individuals spend out of home.

10 As the data do not distinguish between indoor and outdoor locations, we categorized indoor places based on their NAICS code. Whenever the indoor/outdoor nature of the POI can not be inferred from the six-digits code (e.g. “Historical Sites”) or when the NAICS code is unknown, we identify indoor POI evaluating their commercial name.

11 We excluded the census block groups with informational gaps to avoid compositional changes over the sample period.

12 https://www.coronanet-project.org/.

13 We categorized policies as follows. School Closures: we included all policies within the category “School Closures and Regulations” aimed at closing schools. Lockdown: we included all policies in the category “Lockdown”. Masks: we included all policies in the category “Social Distancing” where the word “Mask” appeared in the type subcategory. Those refer to rules on the wearing of masks in public places. Policies related to mask supplying and distribution are thus excluded. Distancing: we included all policies within the category “Curfew”, all policies within the category “Social Distancing” not referring to masks, and policies within the category “Restrictions of Mass Gatherings”. Closure of Businesses or Government Services: we included all policies within the categories “Restriction and Regulation of Businesses” and “Restriction and Regulation of Government Services” aimed at closing these services. We did not consider health resources policies as they are not expected to impact on social activity. We excluded informational campaigns as they are typically introduced to support other policies and thus display very high correlations with the other policies of interest which would result in multicollinearity issues.

14 Our conclusions are very robust to the use of 24-h averages, the inclusion/exclusion of quadratic terms and of interactions between weather variables (results of these robustness tests are not reported in the paper).

15 In our setting, it is reasonable to expect the effect of weather conditions on viral infectiousness to be stronger when social activity is more intense. Equivalently, we expect the effect of social activity to be magnified when weather conditions are more favourable to the spread of the virus.

16 As both the policy interventions and the level of awareness depend to some extent on the evolution of the contagion, one might be concerned about reverse causality. Although policy interventions and social activity are more likely to respond to the evolution of the *number of cases* rather than the *reproduction rate*, possible correlation between the two may complicate the interpretation of our estimates. Yet, both the number of infections and the infection rate can not be immediately observed and can only be estimated retrospectively by looking at the number of cases detected over the following weeks. Indeed, the dependent variable of Equations (1), (3)) can not be observed in *t*, when social activity are determined. What individuals can observe in *t* is the number of deaths and cases confirmed until *t*. As we are controlling for the severity of the epidemiological situation as observable in *t*, the detection lag ensures that causality in Equations (1), (3)) can only go from social activity to the infection rate and not vice versa.

17 As we can directly observe the mediator in Equation (2), these transformations would not be necessary to estimate the effect of weather on social activity (*α*). Yet, the objective of this analysis is not to study the effect of weather on social activity *in general*, but how this mediates the effect of weather on the reproduction rate, which instead can only be estimated with a *transformed* regression.

18 This figure is compatible with the estimated effects of weather on the mediating variable and of the mediator on the reproduction rate. Indeed, the same temperature increase is also predicted to increase social activity by 0.44 SD, which in turn is estimated to increase *R*_0_ by a very similar amount assuming median weather conditions (0.10 according to the estimates in Column 5 of Table A3.

19 Importantly, differently from temperature, wind speed and precipitation may considerably vary within few miles of distance, and hence we might be losing predictive power when aggregating these variables at the CZ level. Furthermore, as with respect to temperature a higher share of their variability originates from high-frequency fluctuations, the statistical power of the analysis on their effects on the reproduction rate is even further impacted by the necessary aggregation in weighted moving averages.

20 Notice that, as the amount of time individuals spend out of home is computed restricting to individuals who spent at least 8 h at their imputed location, extreme caution is required when comparing this estimates with other studies on the effect of stay-at-home orders on the same variable that do not apply the same restriction (Yan et al., 2021, for instance).
