# Supplementary material for: The complex interplay between weather, social activity, and COVID-19 in the US
Source: SSM Popul Health. 2023 May 28;23:101431. doi: 10.1016/j.ssmph.2023.101431 (PMC10225063; doi:10.1016/j.ssmph.2023.101431)
Supplement: Multimedia component 1 [file mmc1.pdf]

Supplementary Material

A1 Modelling the Data Generating Process

In this section, we explain how the equations described in Section 2.2 can be rearranged and expressed as a function of known quantities to estimate the effects of interest on the reproduction rate from the observed number of confirmed cases. The simplifying assumptions that are needed for this purpose are stated and discussed below.

First, we need to rearrange the equations so that the dependent variable is a function of the (observed) confirmed cases rather than the (unobserved) rate of infections. We focus on the equation for the total effect, but the same steps can be easily generalised to the three equations of the mediation model.

$$\text{Total Effect: } \frac{i_{c,t}}{C_{c,t}} = \delta W_{c,t} + \psi X_{c,t} + u_{c,t} \tag{A1}$$

The equation can be equivalently written as follows:

$$i_{c,t} = \delta W_{c,t} C_{c,t} + \psi X_{c,t} C_{c,t} + u'''_{c,t} \tag{A2}$$

Where $u'''_{c,t} \equiv u_{c,t} C_{c,t}$.

Intuitively, the effect of weather conditions on the number of infections is proportional to the number of contagious individuals. Still, as mentioned we do not observe the number of infections nor the number of contagious cases. What we do observe is the daily number of new confirmed cases, $N_{c,t}$, which follows infections with a substantial time-lag and only represents a fraction (ϕ) of the actual number of cases. Importantly, we allow ϕ, the probability of detection, to vary over time and across CZs.

Denoting L_{max} as the maximum possible lag between infection and detection, $\phi_{c,t-j}$ as the probability that a case infected in $t-j$ is ever detected, and p_j as the probability that a case is detected j days after the infection, conditional on that case ever being detected, we can express the expected number of confirmed cases in c, t conditional on the daily number of infections in the previous days as:

$$E(N_{c,t} | i_{c,k}, \forall k < t) = \sum_{j=1}^{L_{max}} \phi_{c,t-j} p_j i_{c,t-j} \tag{A3}$$

Hence, assuming that the actual number of confirmed cases in c, t is well approximated by its expected value:

$$N_{c,t} \approx \sum_{j=1}^{L_{max}} \phi_{c,t-j} p_j i_{c,t-j} \tag{A4}$$

Substituting Equation A2 into Equation A4 and taking the linear parameters out of

the sums, we get:

$$N_{c,t} \approx \delta \sum_{j=1}^{L_{max}} (p_j W_{c,t-j} \phi_{c,t-j} C_{c,t-j}) + \psi \sum_{j=1}^{L_{max}} (p_j X_{c,t-j} \phi_{c,t-j} C_{c,t-j}) + e_{c,t} \tag{A5}$$

Where $e_{c,t} \equiv \sum_{j=1}^{L_{max}} (\phi_{c,t-j} p_j u'''_{c,t-j}) = \sum_{j=1}^{L_{max}} (\phi_{c,t-j} p_j u_{c,t-j} C_{c,t-j})$.

Although apparently complex, the interpretation of Equation A5 is quite intuitive: as infections are not immediately detected, the number of confirmed cases responds to weather conditions (and other factors) with the same time-lag with which infections are detected, and the effect of weather conditions in the previous days is proportional to the corresponding stock of contagious individuals in the area. All the variables on the right-hand side of Equation A2, including the covariates, are thus weighted accordingly.

Then, we normalise Equation A5 dividing both sides by $\sum_{j=1}^{L_{max}} (p_j \phi_{c,t-j} C_{c,t-j})$,[1] and obtain the following regression form:

$$\frac{N_{c,t}}{\sum_{j=1}^{L_{max}} (p_j \phi_{c,t-j} C_{c,t-j})} \approx \delta \frac{\sum_{j=1}^{L_{max}} (p_j W_{c,t-j} \phi_{c,t-j} C_{c,t-j})}{\sum_{j=1}^{L_{max}} (p_j \phi_{c,t-j} C_{c,t-j})} + \psi \frac{\sum_{j=1}^{L_{max}} (p_j X^*_{c,t-j} \phi_{c,t-j} C_{c,t-j})}{\sum_{j=1}^{L_{max}} (p_j \phi_{c,t-j} C_{c,t-j})} + \beta^*_c + \beta^*_t + \varepsilon_{c,t} \tag{A6}$$

Where $\varepsilon_{c,t} \equiv \frac{\sum_{j=1}^{L_{max}} (\phi_{c,t-j} p_j u_{c,t-j} C_{c,t-j})}{\sum_{j=1}^{L_{max}} (p_j \phi_{c,t-j} C_{c,t-j})}$, and $X^*_{c,t-j}$ is the vector of covariates $X_{c,t-j}$ without the fixed effects, and β^*_c and β^*_t are CZ and date fixed effects.

In order to proceed with the estimation of the parameters of interest, we now need to model the probability distribution of the time-lag between infection and confirmation (p_j), and the number of (detected) contagious cases within the CZ in time $\phi_{c,t} C_{c,t}$.

[1] To understand the reason for this final step, consider first the CZ fixed effects β_c included among the regressors $X_{c,t}$ in the original Equation A1, which capture the permanent differences in the level of infectiousness between CZs. This CZ-specific component would enter Equation A5 as follows:

$$\sum_{j=1}^{L_{max}} (p_j \beta_c \phi_{c,t-j} C_{c,t-j})$$

As the values within the sum change with the number of contagious individuals in time, the CZ-specific fixed effect in Equation A1 would not enter Equation A5 as a time-invariant component that can be absorbed through ordinary fixed effects, and its impact on the dependent variable would ultimately depend on the number of contagious individual in time. Yet, being time-invariant, the CZ-specific component can be taken out of the sum, which simplifies after the normalisation. In brief, this normalisation is necessary to absorb the permanent differences in the level of infectiousness between CZs through ordinary fixed effects.

Consider now what happens to the set of date fixed effects β_t instead. As they are not fixed in time, they cannot be taken out of the sum and the same simplification does not apply:

$$\frac{\sum_{j=1}^{L_{max}} (p_j \beta_{t-j} \phi_{c,t-j} C_{c,t-j})}{\sum_{j=1}^{L_{max}} (p_j \phi_{c,t-j} C_{c,t-j})} \equiv \beta^*_{c,t}$$

As the number of contagious individuals in time may vary differently within different CZs, this term may vary both in time and across CZs. Yet, under the assumption that either the time-specific component β_{t-j} or the relative change in the number of contagious individuals in the CZ are enough well-behaved, $\beta^*_{c,t} \approx \beta^*_t$ can still be absorbed with ordinary fixed effects.

Modelling the Detection Delay

To our knowledge, there is no publicly available information on $\hat{p_j}$, the probability distribution of the total number of days from infection to case confirmation.

In the absence of one single source of data reporting information on the dates of infection and confirmation, we combined data from two different sources. First, we estimated the probability distribution of the incubation period (from exposure to symptom onset) from individual data collected by the Infectious Disease Dynamics Group at Johns Hopkins University[2] reporting, for a sample of 181 confirmed cases in China, the time of possible exposure to SARS-CoV-2 and the time of first symptoms onset (Lauer et al., 2020).[3] As the incubation time is a biological parameter, it is not expected to be too context-dependent. We thus took the probability distribution of the incubation period estimated from Chinese cases as a good approximation for the same distribution in the U.S..

Second, we estimated the probability distribution of the time between the onset of symptoms and the reporting of the positive case to the CDC from individual-level data collected from the "COVID-19 case surveillance system database", excluding cases reported before the 1st of April 2020 for higher precision.[4][5]

Assuming statistical independence between the two, we then combined the estimated distribution of the incubation period (Figure A1, left panel) with the estimated distribution of the symptom-to-confirmation time (Figure A1, central panel) to obtain a combined probability distribution of the total lag between infection and confirmation for symptomatic cases (Figure A1, right panel). Assuming that the same distribution can be adopted to model the infection-to-confirmation time-lag for asymptomatic cases, we then fit a Gamma distribution based on the observed first and second sample moments truncating the distribution at 30 days (the 92^{th}percentile) to approximate $\hat{p_j}$.

Figure A1: Incubation Time, Time Between Symptoms and Confirmation, and Total Lag

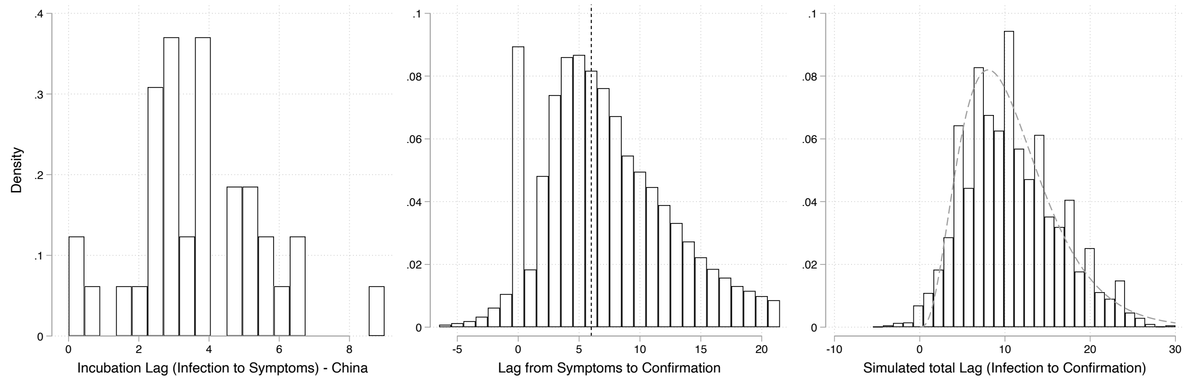

[2]Publicly available at https://github.com/HopkinsIDD/ncov_incubation.

[3]When the exact event date is unknown, the data provide conservative upper and lower bounds for the possible interval of each event.

[4]Publicly available at https://data.cdc.gov/Case-Surveillance/COVID-19-Case-Surveillance-Public-Use-Data/vbim-akqf.

[5]As we have no information on possible lags between the date in which an individual case was reported to the CDC and the date in which the case was included in the official counts (e.g. due to case information validation), we worked under the assumption that this further lag is negligible.

One concern is that improvements in testing or reporting procedures might have resulted in a substantial decrease in the time between infection and confirmation within the sample period, making the adoption of a time-invariant probability distribution problematic for our purposes. Interestingly, despite some fluctuation over time, the median lag between symptoms' onset and case reporting to the CDC in the first and last quarters of the year are identical (7 days).

Modelling the Stock of Contagious Individuals

To estimate the evolution of the number of contagious cases within CZs over time, we first retrospectively reconstructed the daily number of infections, looking at the number of confirmed cases. Then, from the estimated number of daily infections, we estimated the stock of (detected) contagious individuals over time assuming an infectiousness profile.

If $\hat{p}_k$ is the probability that an individual is detected k days after the infection (conditional on the case ever being detected), then we can impute a fraction $\hat{p}_k$ of the cases detected in day t to day $t-k$ to approximate the number of daily infections over time which will eventually be detected ($\widehat{\phi_{c,t} i_{c,t}}$).[6]

Then, we assumed that individuals are infectious from the day after their infection and for the following 15 days (He et al., 2020).[7] Sensitivity checks show that our estimates are quite robust to the choice of alternative infectiousness profiles.

Clearly, unless we are willing to model the probability of detection $\phi_{c,t}$, we cannot estimate the evolution over time of the true number of infections, but only the fraction that is eventually detected through testing. Yet, what we need for the estimation is precisely a proxy of the mass of *detected* contagious individuals over time $\phi_{c,t} C_{c,t}$.

For simplicity, we assumed that $\phi_{c,t-q} \approx \phi_{c,t} \forall q \leq 15$, meaning that the probability of detection is not changing too rapidly and that it is thus approximately constant within the 15 days window in which the individual is assumed to be contagious. This allows to compute an estimate of the stock of (detected) contagious individuals in time as follows:

$$\phi_{c,t} C_{c,t} \approx \widehat{\phi_{c,t} C_{c,t}} = \sum_{q=1}^{15} \widehat{\phi_{c,t-q} i_{c,t-q}} \tag{A7}$$

This simplifying assumption is very convenient as it allows to treat the probability as approximately constant within the sum, making it coincide with the one on the left-hand side of Equation A7, thus freeing us from the need to model the changing probability of detection. However, although the assumption is reasonable when the testing capacity is not changing too rapidly, it may be problematic for the early phases, when the testing capacity, and consequently the probability of detection, were increasing quickly. In practice, this is likely to be a minor issue as our estimation strategy puts little statistical weight on date-CZ cells with few estimated contagious cases. Yet, to make sure that this

[6] Notice that the series of infections is estimated only to approximate the evolution of the stock of contagious individuals over time, and it is not meant to capture high-frequency variations in the actual number of infections. Hence, this measure is not suitable to build daily proxies of infection rates.

[7] Combining the serial interval estimated by He et al. (2020) with the estimated incubation period (see Section A1) and fitting a gamma distribution on the resulting density function, we estimate that 90% of secondary infections occur within this time window.

simplification is not driving our results, in our preferred specification we exclude the first phase of the outbreak.

Final Regression Form

We can now substitute p_j with $\hat{p}_j$ and $\phi_{c,t-j}C_{c,t-j}$ with $\widehat{\phi_{c,t-j}C_{c,t-j}}$ in Equation A6 to obtain the following regression form:

$$\frac{N_{c,t}}{\sum_{j=1}^{L_{max}}(\hat{p}_j\widehat{\phi_{c,t-j}C_{c,t-j}})} \approx \delta\frac{\sum_{j=1}^{L_{max}}(\hat{p}_j W_{c,t-j}\widehat{\phi_{c,t-j}C_{c,t-j}})}{\sum_{j=1}^{L_{max}}(\hat{p}_j\widehat{\phi_{c,t-j}C_{c,t-j}})} + \psi\frac{\sum_{j=1}^{L_{max}}(\hat{p}_j X^*_{c,t-j}\widehat{\phi_{c,t-j}C_{c,t-j}})}{\sum_{j=1}^{L_{max}}(\hat{p}_j\widehat{\phi_{c,t-j}C_{c,t-j}})} + \beta^*_c + \beta^*_t + \varepsilon_{c,t} \tag{A8}$$

Where $\varepsilon_{c,t} \equiv \frac{\sum_{j=1}^{L_{max}}\hat{p}_j u_{c,t-j}\widehat{\phi_{c,t-j}C_{c,t-j}}}{\sum_{j=1}^{L_{max}}(\hat{p}_j\widehat{\phi_{c,t-j}C_{c,t-j}})}$, $X^*_{c,t-j}$ is a set of covariates, and β^*_c and β^*_t are CZ and date fixed effects.

We observe all the terms in Equation A8 except for the linear parameters and the idiosyncratic component $\varepsilon_{c,t}$, and we can thus estimate the parameters of interest in δ fitting the equation through a linear regression where each CZ-day cell is weighted according to the normalisation factor $\sum_{j=1}^{L_{max}}(\hat{p}_j\widehat{\phi_{c,t-j}C_{c,t-j}})$.[8]

The error term $\varepsilon_{c,t}$ enters the equation as a moving average of the original error $u_{c,t}$. Intuitively, a random deviation in daily infectiousness in the CZ will affect the number of confirmed cases for several days, making the error term persistent in time. In estimating Equation A8, we thus cluster the errors at the CZ and date levels to allow for a flexible serial correlation in the error terms over time and in space.

Our estimates can then be converted from effects on the daily reproduction number (the dependent variable in Equation A1) into effects on R_0 (the average number of secondary infections associated with one primary case) multiplying the estimates from the regression by the length of the assumed period of infectiousness.

[8] It is reasonable to assume that the variance of the original error term in the daily infectiousness, $u_{c,t}$, is proportional to the inverse of the number of contagious cases, meaning that when the denominator of the dependent variable in Equation 1 increases, the number of daily infections per contagious individual is less noisy. In practice, this simply means that we are getting more meaningful statistical information from cells with more contagious individuals.

A2 Main Estimates: Regression Tables

Table A1: Estimated Total Effect of Weather on R_0 (δ)

	Estimated effect on R_0			
	1	2	3	4
Temperature	0.01902***	0.00083	0.00110	0.00123
	(0.00454)	(0.00356)	(0.00357)	(0.00370)
Sq. Temperature	-0.00051***	-0.00029***	-0.00030***	-0.00029***
	(0.00009)	(0.00006)	(0.00006)	(0.00006)
Wind	0.07048	-0.04770	-0.04615	-0.04481
	(0.06477)	(0.04641)	(0.04585)	(0.04701)
Sq. Wind	-0.01312	0.00442	0.00421	0.00361
	(0.00834)	(0.00617)	(0.00600)	(0.00608)
Rain	0.48246**	-0.03501	-0.00657	-0.04443
	(0.19217)	(0.14909)	(0.14637)	(0.15072)
Sq. Rain	-0.49616**	0.03822	-0.00282	0.03896
	(0.21036)	(0.17448)	(0.16927)	(0.16989)
Perc. Positive		-0.26024***	-0.25957***	-0.26965***
		(0.01298)	(0.01256)	(0.01306)
Sq. Perc. Positive		0.01339***	0.01327***	0.01386***
		(0.00147)	(0.00144)	(0.00147)
Deaths Past 2 Weeks (Thousands)		-0.03653***	-0.03848***	-0.04967***
		(0.00887)	(0.00870)	(0.01264)
Closure of Businesses or Government Services			0.02610	0.00503
			(0.02448)	(0.03160)
Distancing			-0.04943**	-0.02352
			(0.02212)	(0.02329)
Masks			-0.00162	-0.03424
			(0.02726)	(0.02365)
Lockdown			-0.04470	-0.07291*
			(0.04011)	(0.04324)
School Closure			-0.06644***	-0.07905***
			(0.02300)	(0.02571)
Observations	172,312	172,312	172,312	132,001
Adjusted R-squared	0.222	0.280	0.281	0.254
Mean Dependent Variable	1.11	1.11	1.11	1.10
p-value of F-test on Temp.	0.000***	0.000***	0.000***	0.000***
p-value of F-test on Rain	0.043**	0.973	0.982	0.9460
p-value of F-test on Wind	0.021**	0.254	0.272	0.21
CZ F.E.	Yes	Yes	Yes	Yes
Date F.E.	Yes	Yes	Yes	Yes
Date Restrictions	-	-	-	> May 1st 2020

Notes - *** p<0.01, ** p<0.05, * p<0.1. Robust standard errors clustered both at the CZ and at the date level in parentheses. CZ-day cells are weighted according to the estimated mass of (detected) contagious individuals, and the mean of the dependent variable is computed employing the same statistical weights. Temperature (degrees Celsius) and wind speed (meters per second) are computed as averages over the interval 8 a.m - 10 p.m. (local time). Rain is the fraction of rainy hours (precipitation > 0) over the same interval. Squared variables are indicated with "Sq.". P-values of F-tests refer to joint significance tests for the corresponding weather variable and its interactions. The coefficients of interest of Column 4 are plotted in red in the bottom panel of Figure 3.

Table A2: Estimated Effect of Weather on the Mediator (α)

	Estimated effect on (std) Time Spent Out of Home			
	1	2	3	4
Temperature	0.04229***	0.03804***	0.03823***	0.03851***
	(0.00388)	(0.00381)	(0.00384)	(0.00400)
Sq. Temperature	-0.00084***	-0.00080***	-0.00080***	-0.00079***
	(0.00009)	(0.00009)	(0.00009)	(0.00009)
Wind	0.01653	-0.02628	-0.02597	-0.02214
	(0.04701)	(0.02940)	(0.02853)	(0.02857)
Sq. Wind	-0.01093	-0.00382	-0.00399	-0.00466
	(0.00714)	(0.00425)	(0.00402)	(0.00401)
Rain	0.28224**	0.16351	0.21845**	0.24499**
	(0.11868)	(0.10731)	(0.10629)	(0.10550)
Sq. Rain	-0.42431***	-0.29348**	-0.37512***	-0.42822***
	(0.12918)	(0.12453)	(0.12508)	(0.12533)
Perc. Positive		-0.06758***	-0.06718***	-0.08477***
		(0.01648)	(0.01621)	(0.01767)
Sq. Perc. Positive		0.00617***	0.00607***	0.00725***
		(0.00140)	(0.00137)	(0.00143)
Deaths Past 2 Weeks (Thousands)		-0.03908***	-0.04108***	-0.04995***
		(0.00586)	(0.00521)	(0.00837)
Closure of Businesses or Government Services			0.05461**	0.05390
			(0.02541)	(0.03523)
Distancing			-0.05370*	-0.02735
			(0.02831)	(0.01964)
Masks			0.04431**	-0.01231
			(0.02177)	(0.02024)
Lockdown			-0.13550***	-0.19761***
			(0.03561)	(0.02424)
School Closure			-0.08327***	-0.06595***
			(0.02853)	(0.02498)
Observations	172,312	172,312	172,312	132,001
Adjusted R-squared	0.976	0.978	0.979	0.975
p-value of F-test on Temp.	0.000***	0.000***	0.000***	0.000***
p-value of F-test on Rain	0.000***	0.009***	0.001***	0.000***
p-value of F-test on Wind	0.000***	0.000***	0.000***	0.001***
Mean Dependent Variable	-0.56	-0.56	-0.56	-0.47
CZ F.E.	Yes	Yes	Yes	Yes
Date F.E.	Yes	Yes	Yes	Yes
Date Restrictions	-	-	-	> May 1st 2020

Notes - *** $p<0.01$, ** $p<0.05$, * $p<0.1$. Robust standard errors clustered both at the CZ and at the date level in parentheses. CZ-day cells are weighted according to the estimated mass of (detected) contagious individuals, and the mean of the dependent variable is computed employing the same statistical weights. Temperature (degrees Celsius) and wind speed (meters per second) are computed as averages over the interval 8 a.m - 10 p.m. (local time). Rain is the fraction of rainy hours (precipitation > 0) over the same interval. The mediating variable (Average Time Out of Home) is standardised. Squared variables are indicated with "Sq.". P-values of F-tests refer to joint significance tests for the corresponding weather variable and its interactions. The coefficients of interest of Column 4 are plotted in black in the top panels of Figure 3.

Table A3: Estimated Direct Effect of Weather on R_0 (γ)

	Estimated effect on R_0				
	1	2	3	4	5
Temperature	-0.00275	-0.02014***	-0.02044***	-0.02259***	-0.00844**
	(0.00491)	(0.00385)	(0.00388)	(0.00390)	(0.00359)
Sq. Temperature	-0.00011	0.00030***	0.00030***	0.00039***	-0.00009
	(0.00011)	(0.00010)	(0.00010)	(0.00010)	(0.00006)
Wind	0.03267	-0.02376	-0.02664	-0.00379	-0.04017
	(0.04625)	(0.03387)	(0.03404)	(0.03404)	(0.04413)
Sq. Wind	-0.00519	0.00259	0.00308	-0.00115	0.00489
	(0.00519)	(0.00442)	(0.00442)	(0.00437)	(0.00545)
Rain	0.18787	-0.18716	-0.20655	-0.23916	-0.10277
	(0.21653)	(0.18270)	(0.18250)	(0.18358)	(0.14425)
Sq. Rain	-0.13830	0.26639	0.28396	0.33368	0.14410
	(0.23548)	(0.22976)	(0.22991)	(0.24189)	(0.16409)
Time Out of Home	0.46914***	0.45713***	0.45699***	0.43572***	0.25329***
	(0.09206)	(0.07874)	(0.08003)	(0.08209)	(0.03431)
Sq. Time Out of Home	-0.09860*	0.02622	0.01100	0.02776	0.00210
	(0.05181)	(0.04567)	(0.04779)	(0.06162)	(0.00861)
Wind X Time Out	-0.05496	-0.01654	-0.01795	-0.00872	
	(0.04544)	(0.03729)	(0.03711)	(0.03711)	
Wind X Sq. Time Out	0.01076	-0.01064	-0.00715	-0.03607	
	(0.02297)	(0.01480)	(0.01508)	(0.02262)	
Sq. Wind X Time Out	0.00326	0.00131	0.00138	0.00162	
	(0.00605)	(0.00460)	(0.00457)	(0.00451)	
Sq. Wind X Sq. Time Out	-0.00096	0.00197	0.00152	0.00680**	
	(0.00277)	(0.00217)	(0.00210)	(0.00290)	
Temperature X Time Out	0.00304	-0.01401***	-0.01415***	-0.01686***	
	(0.00370)	(0.00322)	(0.00315)	(0.00317)	
Temperature X Sq. Time Out	0.00242	0.00342	0.00400	0.00747**	
	(0.00336)	(0.00314)	(0.00321)	(0.00288)	
Sq. Temperature X Time Out	-0.00002	0.00031***	0.00031***	0.00037***	
	(0.00011)	(0.00009)	(0.00009)	(0.00009)	
Sq. Temperature X Sq. Time Out	-0.00004	-0.00018**	-0.00019**	-0.00029***	
	(0.00010)	(0.00009)	(0.00009)	(0.00008)	
Rain X Time Out	-0.19508	-0.19920	-0.19969	-0.02503	
	(0.19009)	(0.15761)	(0.15447)	(0.15919)	
Rain X Sq. Time Out	-0.12360	-0.06976	-0.04013	0.13184	
	(0.17088)	(0.12799)	(0.12878)	(0.13396)	
Rain2 X Time Out	0.10779	0.17477	0.18075	-0.02832	
	(0.21398)	(0.18051)	(0.17798)	(0.18308)	
Sq. Rain X Sq. Time Out	0.05095	0.02596	-0.00217	-0.20352	
	(0.18959)	(0.13977)	(0.14043)	(0.15616)	
Perc. Positive		-0.24155***	-0.23960***	-0.25854***	-0.24907***
		(0.01354)	(0.01331)	(0.01332)	(0.01468)
Sq. Perc. Positive		0.01071***	0.01055***	0.01170***	0.01209***
		(0.00145)	(0.00144)	(0.00142)	(0.00152)
Deaths Past 2 Weeks (Thousands)		-0.04111**	-0.04060**	-0.06841***	-0.03794***
		(0.01777)	(0.01655)	(0.01406)	(0.01105)
Closure of Businesses or Government Services			0.01742	0.01065	-0.00882
			(0.02238)	(0.02580)	(0.02696)
Distancing			-0.04142*	-0.00906	-0.01580
			(0.02436)	(0.02293)	(0.02355)
Masks			0.01221	-0.02189	-0.03150
			(0.02311)	(0.02096)	(0.02321)
Lockdown			0.02385	-0.00485	-0.02330
			(0.03180)	(0.03995)	(0.04195)
School Closure			-0.03949*	-0.06094**	-0.06228**
			(0.02077)	(0.02478)	(0.02622)
Observations	172,312	172,312	172,312	132,001	132,001
Adjusted R-squared	0.244	0.289	0.289	0.264	0.259
Mean Dependent Variable	1.11	1.11	1.11	1.10	1.10
Coeff. on Mediator at Median W.	0.387	0.259	0.254	0.23	
Coeff. on Sq. Mediator at Median W.	-0.057	0.01	0.009	0.018	
p-value of F-test on Mediator	0.000***	0.000***	0.000***	0.000***	0.000***
p-value of F-test on Temp.	0.006***	0.000***	0.000***	0.000***	0.000***
p-value of F-test on Rain	0.113	0.538	0.724	0.748	0.64
p-value of F-test on Wind	0.000***	0.532	0.466	0.012**	0.66
CZ F.E.	Yes	Yes	Yes	Yes	Yes
Date F.E.	Yes	Yes	Yes	Yes	Yes
Date Restrictions	-	-	-	$>$ May 1st 2020	$>$ May 1st 2020

Notes - *** p<0.01, ** p<0.05, * p<0.1. Robust standard errors clustered both at the CZ and at the date level in parentheses. CZ-day cells are weighted according to the estimated mass of (detected) contagious individuals, and the mean of the dependent variable is computed employing the same statistical weights. Temperature (degrees Celsius) and wind speed (meters per second) are computed as averages over the interval 8 a.m - 10 p.m. (local time). Rain is the fraction of rainy hours (precipitation > 0) over the same interval. The mediating variable (Average Time Out of Home) is standardised. Squared variables are indicated with "Sq.". P-values of F-tests refer to joint significance tests for the corresponding weather variable and its interactions. The coefficients of interest of Column 4 are plotted in blue in the bottom panel Figure 3 for three different levels of the mediator.

A3 Alternative Mediator: Number of Visits to Indoor Locations

Here, we replicate our main analysis employing as mediating variable an alternative measure of social activity computed as the average number of visits to indoor locations in the CZ-day cell instead of the average amount of time spent out of home in the same cell (see Section 2.1 for more details about this measure and the procedure used to classify indoor places). Results are reported in Tables A4 and A5, and are summarised in Figure A2 for visualization purposes. For the total effects we still refer to estimates in Table A1.

Figure A2: Estimated Total and Direct Effects of Weather on R_0, Alternative Mediator: Number of Visits to Indoor Locations

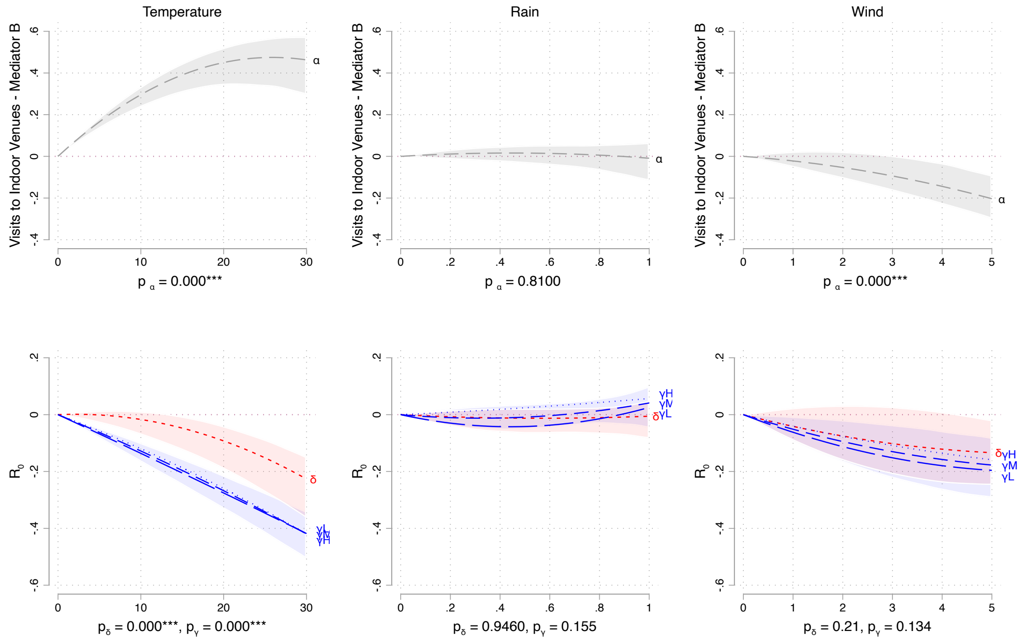

Notes - In the top panels, we plot the estimated effect of each weather variable on the mediator (α). Below, we plot the estimated total effect (δ) in red, together with the direct effect (γ) in blue, holding the level of the mediating variable fixed at three different levels: its mean level (γM), 0.5 SD below (γL), and 0.5 SD above (γH). The mediating variable (Number of Visits to Indoor Locations) is standardised. Each plot reports the corresponding 90% confidence bands, based on 1000 CZ-level block-bootstrap samples. Below each panel, we report the p-value of a joint significance F-test for the corresponding weather variable and its interactions (*** p<0.01, ** p<0.05, * p<0.1). See Column 4 of Tables A1, A4, and A5 for the complete results.

Table A4: Estimated Effect of Weather on the Mediator (α), Alternative Mediating Variable: Number of Visits to Indoor Locations

	Estimated effect on (std) Visits to Indoor Locations			
	1	2	3	4
Temperature	0.03521***	0.03144***	0.03217***	0.03667***
	(0.00402)	(0.00410)	(0.00411)	(0.00413)
Sq. Temperature	-0.00070***	-0.00065***	-0.00065***	-0.00071***
	(0.00011)	(0.00011)	(0.00011)	(0.00011)
Wind	-0.04128	-0.04010	-0.03689	-0.01733
	(0.03297)	(0.03025)	(0.02891)	(0.03044)
Sq. Wind	-0.00172	-0.00118	-0.00162	-0.00466
	(0.00404)	(0.00357)	(0.00335)	(0.00358)
Rain	0.13964	0.10198	0.14379	0.07220
	(0.11300)	(0.11117)	(0.11418)	(0.11357)
Sq. Rain	-0.11117	-0.06628	-0.14304	-0.08107
	(0.12534)	(0.12751)	(0.12922)	(0.13282)
Perc. Positive		-0.09567***	-0.09186***	-0.10863***
		(0.02619)	(0.02524)	(0.02371)
Sq. Perc. Positive		0.00808**	0.00760**	0.00914***
		(0.00314)	(0.00306)	(0.00292)
Deaths Past 2 Weeks (Thousands)		0.00126	-0.00087	0.00780
		(0.00813)	(0.00646)	(0.00846)
Closure of Businesses or Government Services			0.01612	-0.02692
			(0.04217)	(0.04630)
Distancing			-0.04980	-0.01157
			(0.03152)	(0.04315)
Masks			0.01213	-0.00409
			(0.03763)	(0.04409)
Lockdown			-0.14172***	-0.19493***
			(0.04197)	(0.04038)
School Closure			-0.08222***	-0.01791
			(0.02652)	(0.02377)
Observations	160,536	160,536	160,536	120,321
Adjusted R-squared	0.932	0.934	0.935	0.931
p-value of F-test on Temp.	0.000***	0.000***	0.000***	0.000***
p-value of F-test on Rain	0.447	0.586	0.453	0.8100
p-value of F-test on Wind	0.000***	0.001***	0.001***	0.000***
Mean Dependent Variable	-0.09	-0.09	-0.09	-0.02
CZ F.E.	Yes	Yes	Yes	Yes
Date F.E.	Yes	Yes	Yes	Yes
Date Restrictions	-	-	-	> May 1st 2020

Notes - *** p<0.01, ** p<0.05, * p<0.1. Robust standard errors clustered both at the CZ and at the date level in parentheses. CZ-day cells are weighted according to the estimated mass of (detected) contagious individuals, and the mean of the dependent variable is computed employing the same statistical weights. Temperature (degrees Celsius) and wind speed (meters per second) are computed as averages over the interval 8 a.m - 10 p.m. (local time). Rain is the fraction of rainy hours (precipitation > 0) over the same interval. The mediating variable (Number of Visits to Indoor Locations) is standardised. Squared variables are indicated with "Sq.". P-values of F-tests refer to joint significance tests for the corresponding weather variable and its interactions. The coefficients of interest of Column 4 are plotted in black in the top panels of Figure A2.

Table A5: Estimated Direct Effect of Weather on R_0 (γ), Alternative Mediating Variable: Number of Visits to Indoor Locations

	Estimated effect on R_0				
	1	2	3	4	5
Temperature	-0.00470	-0.01248***	-0.01174***	-0.01219***	-0.01414***
	(0.00519)	(0.00422)	(0.00426)	(0.00436)	(0.00370)
Sq. Temperature	-0.00015	-0.00003	-0.00005	-0.00006	0.00000
	(0.00012)	(0.00009)	(0.00009)	(0.00010)	(0.00008)
Wind	-0.03700	-0.05073	-0.05165	-0.05524	-0.08483**
	(0.04286)	(0.03353)	(0.03456)	(0.03520)	(0.03609)
Sq. Wind	0.00235	0.00444	0.00454	0.00396	0.00851**
	(0.00513)	(0.00390)	(0.00397)	(0.00397)	(0.00411)
Rain	-0.02928	-0.11654	-0.08655	-0.07627	-0.20309
	(0.17776)	(0.16407)	(0.15799)	(0.15922)	(0.15400)
Sq. Rain	0.04836	0.17896	0.13700	0.11741	0.22731
	(0.18863)	(0.19298)	(0.18497)	(0.18609)	(0.17765)
Visits to Indoor Places	0.10056	0.07197	0.06883	0.07068	0.17126***
	(0.09576)	(0.08813)	(0.08711)	(0.09030)	(0.03229)
Sq. Visits to Indoor Places	-0.03113	0.05027	0.04887	0.05053	-0.02843***
	(0.04445)	(0.03883)	(0.03889)	(0.04416)	(0.00780)
Wind X Visits Indoor	0.01627	0.04594	0.03787	0.03911	
	(0.04516)	(0.03847)	(0.03804)	(0.04323)	
Wind X Sq. Visits Indoor	-0.02462	-0.01922	-0.01771	-0.02861	
	(0.02156)	(0.01639)	(0.01653)	(0.01804)	
Sq. Wind X Visits Indoor	0.00060	-0.00660	-0.00570	-0.00564	
	(0.00627)	(0.00481)	(0.00480)	(0.00539)	
Wind2 X Sq. Visits Indoor	-0.00029	0.00149	0.00133	0.00434	
	(0.00389)	(0.00279)	(0.00279)	(0.00286)	
Temperature X Visits Indoor	0.00514	0.00184	0.00262	0.00428	
	(0.00584)	(0.00405)	(0.00398)	(0.00426)	
Temperature X Sq. Visits Indoor	0.00101	-0.00253	-0.00281	-0.00354	
	(0.00360)	(0.00221)	(0.00219)	(0.00282)	
Sq. Temperature X Visits Indoor	-0.00004	-0.00004	-0.00007	-0.00015	
	(0.00016)	(0.00013)	(0.00012)	(0.00012)	
Sq. Temperature X Sq. Visits Indoor	0.00004	0.00007	0.00009	0.00012	
	(0.00010)	(0.00006)	(0.00006)	(0.00008)	
Rain X Visits Indoor	0.54206*	0.19589	0.18690	0.34753	
	(0.27849)	(0.21930)	(0.21689)	(0.22970)	
Rain X Sq. Visits Indoor	-0.27000**	-0.18166*	-0.18524*	-0.23023**	
	(0.11006)	(0.09839)	(0.09662)	(0.10518)	
Rain2 X Visits Indoor	-0.42663	-0.14453	-0.11167	-0.29734	
	(0.30517)	(0.22931)	(0.22629)	(0.23164)	
Rain2 X Sq. Visits Indoor	0.23871*	0.13825	0.13691	0.19266*	
	(0.12543)	(0.10691)	(0.10439)	(0.11099)	
Perc. Positive		-0.28802***	-0.28703***	-0.30050***	-0.30420***
		(0.02046)	(0.02000)	(0.02015)	(0.02079)
Sq. Perc. Positive		0.02022***	0.02006***	0.02077***	0.02133***
		(0.00298)	(0.00293)	(0.00297)	(0.00307)
Deaths Past 2 Weeks (Thousands)		-0.03177***	-0.03199***	-0.04740***	-0.04646***
		(0.00899)	(0.00857)	(0.00866)	(0.00900)
Closure of Businesses or Government Services			0.02999	0.02114	0.01351
			(0.02120)	(0.02709)	(0.02710)
Distancing			-0.03390	-0.01264	-0.01583
			(0.02113)	(0.02407)	(0.02348)
Masks			0.00863	-0.02783	-0.02547
			(0.02883)	(0.02707)	(0.02578)
Lockdown			-0.04040	-0.09764**	-0.08696**
			(0.03885)	(0.04221)	(0.04049)
School Closure			-0.05216***	-0.07738***	-0.07337***
			(0.01843)	(0.02283)	(0.02279)
Observations	160,536	160,536	160,536	120,321	120,321
Adjusted R-squared	0.251	0.288	0.288	0.256	0.255
Mean Dependent Variable	1.11	1.11	1.11	1.10	1.10
Coeff. on Mediator at Median W.	0.267	0.181	0.166	0.18	
Coeff. on Sq. Mediator at Median W.	-0.08	-0.023	-0.022	-0.028	
p-value of F-test on Mediator	0.000***	0.000***	0.000***	0.000***	0.000***
p-value of F-test on Temp.	0.001***	0.000***	0.000***	0.000***	0.000***
p-value of F-test on Rain	0.102	0.131	0.099*	0.155	0.42
p-value of F-test on Wind	0.048**	0.233	0.254	0.134	0.054*
CZ F.E.	Yes	Yes	Yes	Yes	Yes
Date F.E.	Yes	Yes	Yes	Yes	Yes
Date Restrictions	-	-	-	> May 1st 2020	> May 1st 2020

Notes - *** p<0.01, ** p<0.05, * p<0.1. Robust standard errors clustered both at the CZ and at the date level in parentheses. CZ-day cells are weighted according to the estimated mass of (detected) contagious individuals, and the mean of the dependent variable is computed employing the same statistical weights. Temperature (degrees Celsius) and wind speed (meters per second) are computed as averages over the interval 8 a.m - 10 p.m. (local time). Rain is the fraction of rainy hours (precipitation > 0) over the same interval. The mediating variable (Number of Visits to Indoor Locations) is standardised. Squared variables are indicated with "Sq.". P-values of F-tests refer to joint significance tests for the corresponding weather variable and its interactions. The coefficients of interest of Column 4 are plotted in blue in the bottom panel of Figure A2 for three different levels of the mediator.

A4 Heterogeneous Effect Analysis by COVID-19 Incidence

We replicate our main estimates splitting the sample according to the number of confirmed cases in the previous two weeks (computed as a share of the population in the cell) to investigate possible heterogeneity of the effects by COVID-19 incidence. Results are reported in Tables A6, A7 and A8, and summarised in Figure A3 for visualization purposes.

Table A6: Estimated Total Effect of Weather on R_0 (δ), Heterogenous Effects by Local COVID-19 Incidence

	Estimated effect on R_0		
	All	Low Incidence	High Incidence
Temperature	0.00123	0.00495	-0.01282***
	(0.00370)	(0.00565)	(0.00341)
Sq. Temperature	-0.00029***	-0.00035***	-0.00020***
	(0.00006)	(0.00010)	(0.00007)
Wind	-0.04481	-0.03700	0.00574
	(0.04701)	(0.05643)	(0.03125)
Sq. Wind	0.00361	0.00185	-0.00216
	(0.00608)	(0.00693)	(0.00343)
Rain	-0.04443	0.10837	-0.31904*
	(0.15072)	(0.19143)	(0.18900)
Sq. Rain	0.03896	-0.07748	0.21791
	(0.16989)	(0.24026)	(0.20389)
Perc. Positive	-0.26965***	-0.33603***	-0.29375***
	(0.01306)	(0.02618)	(0.01370)
Sq. Perc. Positive	0.01386***	0.02522***	0.01239***
	(0.00147)	(0.00410)	(0.00107)
Deaths Past 2 Weeks (Thousands)	-0.04967***	-0.04340***	-0.37183
	(0.01264)	(0.01238)	(0.22864)
Closure of Businesses or Government Services	0.00503	0.03389	-0.05631
	(0.03160)	(0.03570)	(0.04773)
Distancing	-0.02352	0.00377	-0.00791
	(0.02329)	(0.03053)	(0.03608)
Masks	-0.03424	-0.03966	-0.05050*
	(0.02365)	(0.03295)	(0.03039)
Lockdown	-0.07291*	-0.08389*	-0.12601
	(0.04324)	(0.04647)	(0.12250)
School Closure	-0.07905***	-0.10065***	0.02047
	(0.02571)	(0.02308)	(0.04457)
Observations	132,001	47,993	84,005
Adjusted R-squared	0.254	0.345	0.201
Mean Dependent Variable	1.10	1.11	1.08
p-value of F-test on Temp.	0.000***	0.000***	0.000***
p-value of F-test on Rain	0.9460	0.663	0.043**
p-value of F-test on Wind	0.21	0.259	0.556
CZ F.E.	Yes	Yes	Yes
Date F.E.	Yes	Yes	Yes
Date Restrictions	$>$ May 1st 2020	$>$ May 1st 2020	$>$ May 1st 2020

Notes - *** p<0.01, ** p<0.05, * p<0.1. Robust standard errors clustered both at the CZ and at the date level in parentheses. CZ-day cells are weighted according to the estimated mass of (detected) contagious individuals, and the mean of the dependent variable is computed employing the same statistical weights. Temperature (degrees Celsius) and wind speed (meters per second) are computed as averages over the interval 8 a.m - 10 p.m. (local time). Rain is the fraction of rainy hours (precipitation > 0) over the same interval. Squared variables are indicated with "Sq.". P-values of F-tests refer to joint significance tests for the corresponding weather variable and its interactions. Low (high) Incidence: number of confirmed cases in the previous two weeks as a share of the population below (above) its median level (4 cases per $100,000$ inhabitants). The coefficients of interest of Column 2 and 3 are plotted in red in the bottom of panels (a) and (b) of Figure A3.

Table A7: Estimated Effect of Weather on the Mediator (α), Heterogenous Effects by Local COVID-19 Incidence

	Estimated effect on (std) Time Spent Out of Home		
	All	Low Incidence	High Incidence
Temperature	0.03851***	0.04907***	0.02058***
	(0.00400)	(0.00564)	(0.00252)
Sq. Temperature	-0.00079***	-0.00103***	-0.00034***
	(0.00009)	(0.00013)	(0.00008)
Wind	-0.02214	-0.01832	0.02902
	(0.02857)	(0.03468)	(0.02717)
Sq. Wind	-0.00466	-0.00458	-0.01193***
	(0.00401)	(0.00398)	(0.00333)
Rain	0.24499**	0.22803**	0.08755
	(0.10550)	(0.11106)	(0.12243)
Sq. Rain	-0.42822***	-0.35981***	-0.37078***
	(0.12533)	(0.12645)	(0.13451)
Perc. Positive	-0.08477***	-0.08046**	-0.09956***
	(0.01767)	(0.04047)	(0.01464)
Sq. Perc. Positive	0.00725***	0.01048**	0.00789***
	(0.00143)	(0.00464)	(0.00118)
Deaths Past 2 Weeks (Thousands)	-0.04995***	-0.04834***	-0.17386
	(0.00837)	(0.00988)	(0.21329)
Closure of Businesses or Government Services	0.05390	0.09396***	-0.05586
	(0.03523)	(0.03435)	(0.05440)
Distancing	-0.02735	-0.05386**	0.02857
	(0.01964)	(0.02637)	(0.02960)
Masks	-0.01231	-0.00101	-0.00833
	(0.02024)	(0.03007)	(0.02166)
Lockdown	-0.19761***	-0.19827***	-0.20651***
	(0.02424)	(0.02401)	(0.07713)
School Closure	-0.06595***	-0.08697***	-0.05143
	(0.02498)	(0.02672)	(0.05318)
Observations	132,001	47,993	84,005
Adjusted R-squared	0.975	0.973	0.969
p-value of F-test on Temp.	0.000***	0.000***	0.000***
p-value of F-test on Rain	0.000***	0.007***	0.000***
p-value of F-test on Wind	0.001***	0.000***	0.000***
Mean Dependent Variable	-0.47	-0.75	0.009
CZ F.E.	Yes	Yes	Yes
Date F.E.	Yes	Yes	Yes
Date Restrictions	> May 1st 2020	> May 1st 2020	> May 1st 2020

Notes - *** p<0.01, ** p<0.05, * p<0.1. Robust standard errors clustered both at the CZ and at the date level in parentheses. CZ-day cells are weighted according to the estimated mass of (detected) contagious individuals, and the mean of the dependent variable is computed employing the same statistical weights. Temperature (degrees Celsius) and wind speed (meters per second) are computed as averages over the interval 8 a.m - 10 p.m. (local time). Rain is the fraction of rainy hours (precipitation > 0) over the same interval. The mediating variable (Average Time Out of Home) is standardised. Squared variables are indicated with "Sq.". P-values of F-tests refer to joint significance tests for the corresponding weather variable and its interactions. Low (high) Incidence: number of confirmed cases in the previous two weeks as a share of the population below (above) its median level (4 cases per $100,000$ inhabitants). The coefficients of interest of Column 2 and 3 are plotted in black in the top of panels (a) and (b) of Figure A3.

Table A8: Estimated Direct Effect of Weather on R_0 (γ), Heterogenous Effects by Local COVID-19 Incidence

	Estimated effect on R_0		
	All	Low Incidence	High Incidence
Temperature	-0.02259***	-0.04063***	-0.02361***
	(0.00390)	(0.00858)	(0.00417)
Sq. Temperature	0.00039***	0.00087***	0.00009
	(0.00010)	(0.00021)	(0.00011)
Wind	-0.00379	0.01703	-0.01112
	(0.03404)	(0.04039)	(0.03897)
Sq. Wind	-0.00115	-0.00664	0.00010
	(0.00437)	(0.00508)	(0.00460)
Rain	-0.23916	-0.15162	-0.05486
	(0.18358)	(0.28299)	(0.19226)
Sq. Rain	0.33368	0.26728	-0.02185
	(0.24189)	(0.37520)	(0.21560)
Time Out of Home	0.43572***	0.82879***	0.28852***
	(0.08209)	(0.22543)	(0.07427)
Sq. Time Out of Home	0.02776	0.20585	-0.06247
	(0.06162)	(0.15873)	(0.07087)
Wind X Time Out	-0.00872	-0.12915	0.01614
	(0.03711)	(0.10727)	(0.03053)
Wind X Sq. Time Out	-0.03607	-0.12729**	-0.01724
	(0.02262)	(0.06349)	(0.03109)
Sq. Wind X Time Out	0.00162	0.01899	-0.00012
	(0.00451)	(0.01573)	(0.00393)
Sq. Wind X Sq. Time Out	0.00680**	0.02208**	0.00206
	(0.00290)	(0.00981)	(0.00354)
Temperature X Time Out	-0.01686***	-0.02929**	-0.02395***
	(0.00317)	(0.01386)	(0.00396)
Temperature X Sq. Time Out	0.00747**	0.00526	0.01121***
	(0.00288)	(0.00861)	(0.00352)
Sq. Temperature X Time Out	0.00037***	0.00058*	0.00055***
	(0.00009)	(0.00035)	(0.00011)
Sq. Temperature X Sq. Time Out	-0.00029***	-0.00034	-0.00023**
	(0.00008)	(0.00022)	(0.00010)
Rain X Time Out	-0.02503	0.05670	0.29047
	(0.15919)	(0.32454)	(0.20380)
Rain X Sq. Time Out	0.13184	0.17943	-0.32339
	(0.13396)	(0.20792)	(0.19581)
Rain2 X Time Out	-0.02832	-0.07285	-0.42718**
	(0.18308)	(0.38103)	(0.21414)
Sq. Rain X Sq. Time Out	-0.20352	-0.24139	0.34009
	(0.15616)	(0.25251)	(0.21335)
Perc. Positive	-0.25854***	-0.31615***	-0.27772***
	(0.01332)	(0.02710)	(0.01316)
Sq. Perc. Positive	0.01170***	0.02277***	0.01085***
	(0.00142)	(0.00383)	(0.00111)
Deaths Past 2 Weeks (Thousands)	-0.06841***	-0.11135***	-0.20617
	(0.01406)	(0.02604)	(0.17912)
Closure of Businesses or Government Services	0.01065	0.02508	-0.04387
	(0.02580)	(0.03524)	(0.04711)
Distancing	-0.00906	0.01122	-0.01004
	(0.02293)	(0.02906)	(0.03686)
Masks	-0.02189	-0.02572	-0.04190
	(0.02096)	(0.03187)	(0.02996)
Lockdown	-0.00485	0.01331	-0.08567
	(0.03995)	(0.03808)	(0.11401)
School Closure	-0.06094**	-0.06432***	0.03676
	(0.02478)	(0.02380)	(0.04320)
Observations	132,001	47,993	84,005
Adjusted R-squared	0.264	0.358	0.206
Mean Dependent Variable	1.10	1.11	1.08
Coeff. on Mediator at Median W.	0.23	0.273	0.069
Coeff. on Sq. Mediator at Median W.	0.018	0.004	0.038
p-value of F-test on Mediator	0.000***	0.000***	0.000***
p-value of F-test on Temp.	0.000***	0.000***	0.000***
p-value of F-test on Rain	0.748	0.9430	0.108
p-value of F-test on Wind	0.012**	0.001***	0.8100
CZ F.E.	Yes	Yes	Yes
Date F.E.	Yes	Yes	Yes
Date Restrictions	> May 1st 2020	> May 1st 2020	> May 1st 2020

Notes - *** p<0.01, ** p<0.05, * p<0.1. Robust standard errors clustered both at the CZ and at the date level in parentheses. CZ-day cells are weighted according to the estimated mass of (detected) contagious individuals, and the mean of the dependent variable is computed employing the same statistical weights. Temperature (degrees Celsius) and wind speed (meters per second) are computed as averages over the interval 8 a.m - 10 p.m. (local time). Rain is the fraction of rainy hours (precipitation > 0) over the same interval. The mediating variable (Average Time Out of Home) is standardised. Squared variables are indicated with "Sq.". P-values of F-tests refer to joint significance tests for the corresponding weather variable and its interactions. Low (high) Incidence: number of confirmed cases in the previous two weeks as a share of the population below (above) its median level (4 cases per 100,000 inhabitants). The coefficients of interest of Column 2 and 3 are plotted in blue in the bottom of panels (a) and (b) of Figure A3 for three different levels of the mediator.

Figure A3: Heterogeneous Effect Analysis by COVID-19 Incidence

a) Low COVID-19 Incidence

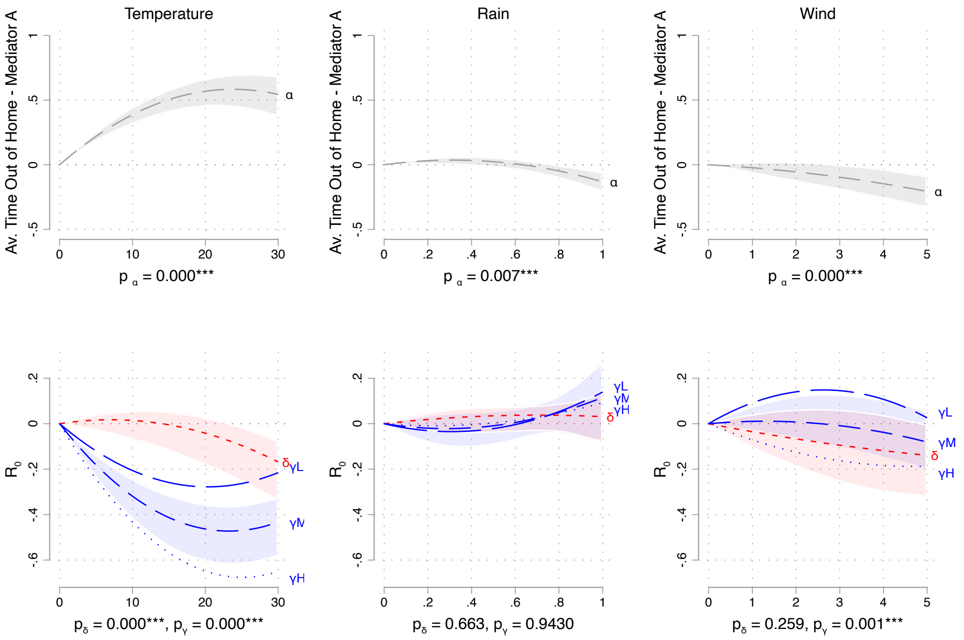

b) High COVID-19 Incidence

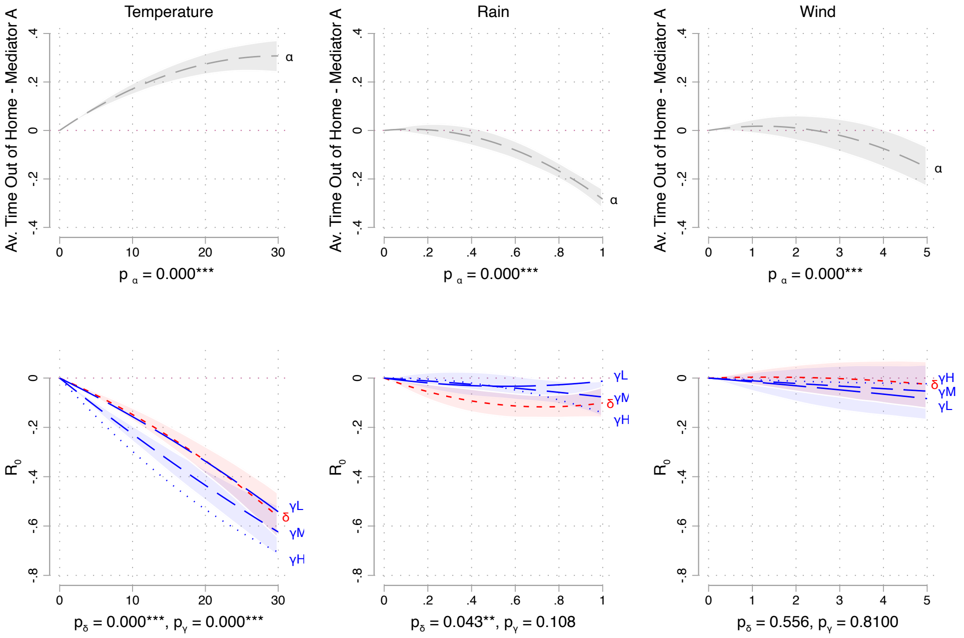

Notes - Low (high) COVID-19 Incidence: number of confirmed cases in the previous two weeks as a share of the population below (above) the median level (4 cases per $100,000$ inhabitants). In the top panels, we plot the estimated effect of each weather variable on the mediator (α). Below, we plot the estimated total effect (δ) in red, together with the direct effect (γ) in blue, holding the level of the mediating variable (Average Time Out of Home) fixed at three different levels: its mean level (γM), 0.5 SD below (γL), and 0.5 SD above (γH). Each plot reports the corresponding 90% confidence bands, based on 1000 CZ-level block-bootstrap samples. Below each panel, we report the p-value of a joint significance F-test for the corresponding weather variable and its interactions (*** p<0.01, ** p<0.05, * p<0.1). See Tables A6, A7, and A8.

A5 Further Robustness Tests

Robustness Test 1: Alternative Infectiousness Profile

In this first robustness test, we replicate the main results assuming an alternative infectiousness profile. In particular, when modelling the stock of contagious individuals from the number of infections, instead of assuming that an individual is contagious from the day after his/her infection and for the following 15 days as in our main analysis, we employ the serial interval profile estimated by He et al. (2020) to weight each positive case by the corresponding probability of generating a secondary infection in the following days. Our conclusions, summarised in Figure A4, are very robust to this change.

Figure A4: Estimated Total and Direct Effects of Weather on R_0, Alternative Infectiousness Profile

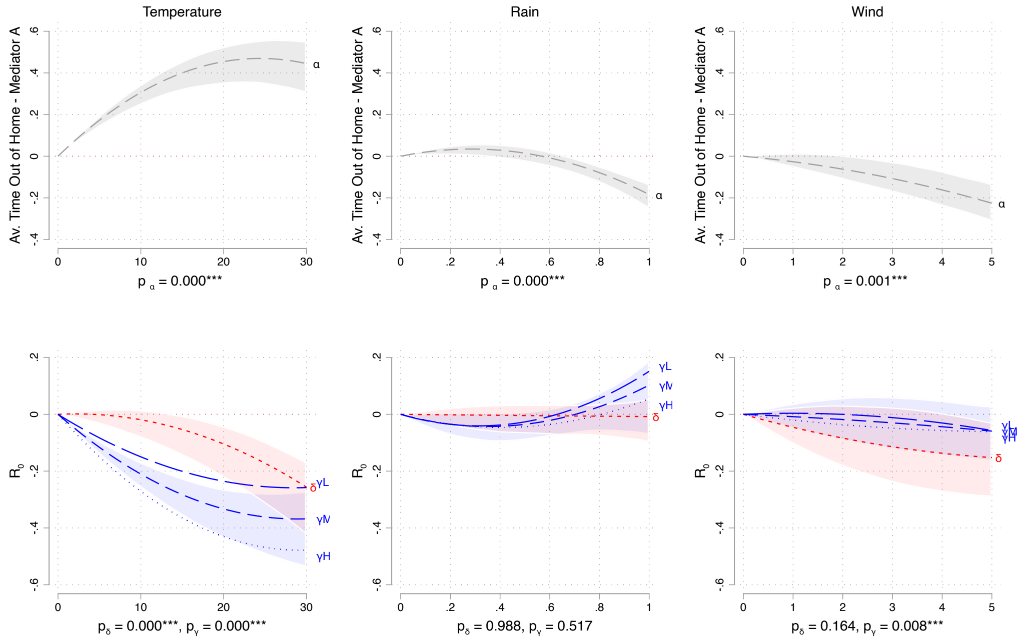

Notes - In the top panels, we plot the estimated effect of each weather variable on the mediator (α). Below, we plot the estimated total effect (δ) in red, together with the direct effect (γ) in blue, holding the level of the mediating variable fixed at three different levels: its mean level (γM), 0.5 SD below (γL), and 0.5 SD above (γH). The mediating variable (Average Time Out of Home) is standardised. Each plot reports the corresponding 90% confidence bands, based on 1000 CZ-level block-bootstrap samples. Below each panel, we report the p-value of a joint significance F-test for the corresponding weather variable and its interactions (*** p<0.01, ** p<0.05, * p<0.1).

Robustness Test 2: Confirmed Cases as Moving Average

In this second robustness test, we replicate our main results employing a 7-day moving average of the number of confirmed cases instead of the raw count ($N_{c,t}$). As Figure A5 shows, our results are not sensitive to this correction. Hence, we conclude that they are not driven by anomalies or outliers in the data.

Figure A5: Estimated Total and Direct Effects of Weather on R_0, Confirmed Cases as Moving Average

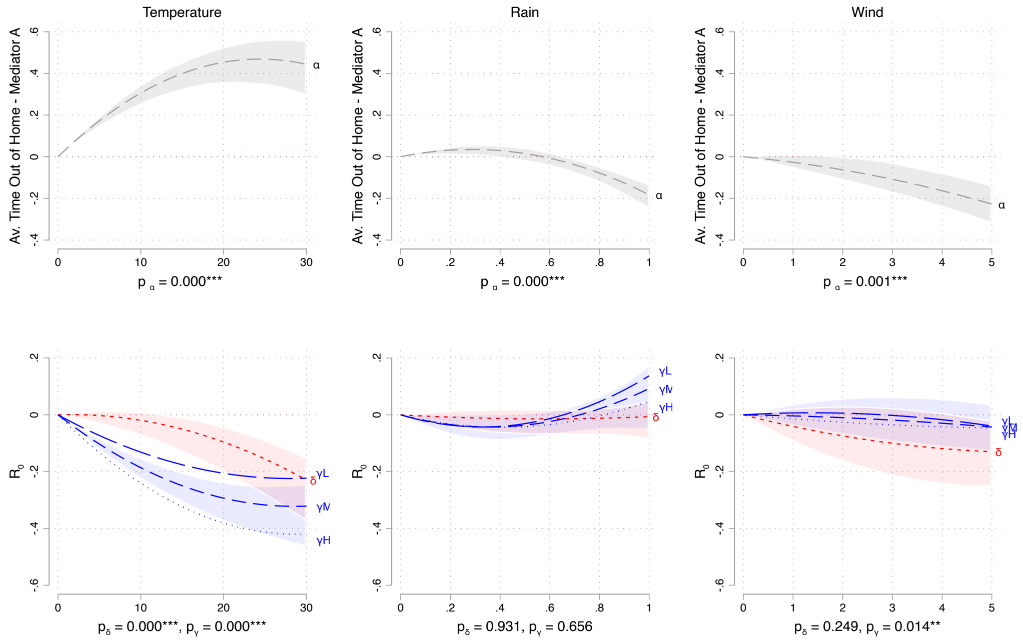

Notes - In the top panels, we plot the estimated effect of each weather variable on the mediator (α). Below, we plot the estimated total effect (δ) in red, together with the direct effect (γ) in blue, holding the level of the mediating variable fixed at three different levels: its mean level (γM), 0.5 SD below (γL), and 0.5 SD above (γH). The mediating variable (Average Time Out of Home) is standardised. Each plot reports the corresponding 90% confidence bands, based on 1000 CZ-level block-bootstrap samples. Below each panel, we report the p-value of a joint significance F-test for the corresponding weather variable and its interactions (*** p<0.01, ** p<0.05, * p<0.1).

References

He, X., E. H. Y. Lau, P. Wu, X. Deng, J. Wang, X. Hao, Y. C. Lau, J. Y. Wong, Y. Guan, X. Tan, X. Mo, Y. Chen, B. Liao, W. Chen, F. Hu, Q. Zhang, M. Zhong, Y. Wu, L. Zhao, F. Zhang, B. J. Cowling, F. Li, and G. M. Leung (2020). Temporal dynamics in viral shedding and transmissibility of COVID-19. *Nature Medicine 26*. (Cited on pages 4 and 16.)

Lauer, S. A., K. H. Grantz, Q. Bi, F. K. Jones, Q. Zheng, H. R. Meredith, A. S. Azman, N. G. Reich, and J. Lessler (2020). The incubation period of coronavirus disease 2019 (COVID-19) from publicly reported confirmed cases: Estimation and application. *Annals of Internal Medicine 172*(9), 577–582. (Cited on page 3.)
